# Understanding changes to children's connection to nature during the COVID‐19 pandemic and implications for child well‐being

**DOI:** 10.1002/pan3.10270

**Published:** 2021-10-13

**Authors:** Samantha Friedman, Susan Imrie, Elian Fink, Mina Gedikoglu, Claire Hughes

**Affiliations:** ^1^ Centre for Family Research University of Cambridge Cambridge UK; ^2^ School of Psychology University of Sussex Sussex UK

**Keywords:** child well‐being, children, connection to nature, COVID‐19 pandemic, pro‐environmental behaviour

## Abstract

1. While psychological connection to nature is known to be associated with both pro‐environmental behaviours and well‐being, there is an urgent need to extend this research to consider impacts from the COVID‐19 lockdown period. Examining whether children's connection to nature changed during this period, identifying the drivers of these changes and determining the links between connection to nature and child well‐being can each serve to guide post‐lockdown initiatives to promote children's connection to nature.
2. Three findings emerged from this UK sample of 376 families with young children. First, nearly two thirds of parents reported a change (most typically, an increase) in their child's connection to nature. Explanations for this increase included having more time, increased enjoyment of nature and increased awareness or interest in nature. Second, a third of children whose connection to nature decreased during the pandemic displayed increased problems of well‐being—manifest as either ‘acting out’ (externalising problems) or sadness/anxiety (internalising problems). Third, an increase in connection to nature during the pandemic was more evident for children from affluent families than for their less affluent peers.
3. While connecting to nature may be an effective means of addressing child problems of well‐being, the divergent findings for children from different family backgrounds indicate that efforts to enhance connection to nature should focus on the barriers experienced by children from less affluent families.

A free Plain Language Summary can be found within the Supporting Information of this article.

## Abstract 2

A free Plain Language Summary can be found within the Supporting Information of this article.

## 1 INTRODUCTION

Alongside its devastating health effects, the COVID‐19 pandemic has led to global disruptions to family life, with young children at especially elevated risk of long‐term negative consequences (Benner & Mistry, 2020; Bignardi et al., 2020; National Health Service, 2020). Prior studies of early risk and resilience demonstrate that protective factors straddle many different levels—from individual child characteristics (e.g. Barnard, 1994; Bolger & Patterson, 2001; Yule et al., 2019) to supportive family relationships (e.g. Prime et al., 2020; Taraban & Shaw, 2018) and important, but often overlooked, wider contextual factors, including children's cultural and physical environments (e.g. Ungar, 2011). The current UK‐based study addressed this third level via a focus on children's connection to nature.

Connection to nature is defined as, ‘the extent to which an individual includes nature within his/her cognitive representation of self’ (Schultz, 2002, p. 67). As a concept, it builds upon access to nature, or simply having green space in reasonable proximity to the family's residence or the child's educational setting, and engagement with nature, or time spent physically in that green space (White et al., 2019). While very stressful for many families, the early stages of the pandemic also gave some children in the United Kingdom new opportunities to connect with nature. In particular, the first lockdown (26 March–4 July 2020) coincided with a period of beautiful spring weather in the United Kingdom that prompted many families to enjoy time in their gardens or to take a government‐sanctioned daily walk. In addition, empty roads, improved air quality and the sudden drop in noise and light pollution enabled birds, plants and animals to thrive in previously inhospitable environments, while also providing new opportunities for children to listen to birdsong or notice wildlife (European Environmental Agency, 2020; Khan et al., 2020; National Centre for Atmospheric Science, 2020; Oliver, 2021; Rume & Didar‐Ul Islam, 2020; Zambrano‐Monserrate et al., 2020).

Arguably, this connection to nature may have helped to buffer some children against the adverse consequences associated with the sudden loss of school activities, routines and social interactions. Access to and engagement with green space are each associated with wide‐ranging benefits for children (and adults), including reductions in stress (Wells & Evans, 2003), improvements in emotional and behavioural problems (Richardson et al., 2017; Vanaken & Danckaerts, 2018) and lower levels of anxiety and depression (Maas et al., 2009). Access to residential green space has been associated with benefits to well‐being in young children (Andrusaityte et al., 2020; Feng & Astell‐Burt, 2017) and adolescents (Ward et al., 2016). In a survey of Japanese adults conducted during the pandemic, those with more frequent green space use and views of green space from their windows reported decreased levels of depression and anxiety and increased levels of subjective happiness suggesting that nature can serve a mitigating role in some of the negative mental health implications resulting from the pandemic (Soga et al., 2020).

Beyond access, developing and sustaining a psychological connection to nature is also important. On the one hand, greater connection to nature is associated with fewer behavioural problems in younger children (Sobko et al., 2018) and with greater life satisfaction in adolescents (Richardson et al., 2015). Even light‐touch, school‐based programs that increase connection to nature (e.g.those involving just 1 hour a week) also produce long‐lasting improvements to child mood and well‐being, as compared with ratings for children in a treatment‐as‐usual control group who did not take part in a biodiversity‐focussed outdoor program (Harvey et al., 2020). Conversely, given the increasing mental health problems experienced by children and young people (Deighton et al., 2019; Terhaag et al., 2021; Waite et al., 2021), it is notable that several lines of evidence indicate a growing disconnection between humans, specifically children, and the natural world (Balmford et al., 2002; Bragg et al., 2013; Moss, 2012; Natural England, 2009).

This disconnection is particularly important when considered alongside stress recovery theory (Ulrich et al., 1991), which suggests that exposure to natural settings accelerates recovery from stressful stimuli. Decreased access to, engagement with and connection to nature also means children are missing out on the well‐being‐related benefits afforded by nature. This is especially relevant in the pandemic context, with studies showing that lockdown and associated changes to normal routine and separation from peers and extended family have exacerbated mental health problems in young people (Bignardi et al., 2020; National Health Service, 2020).

A disconnection from nature may reflect barriers that limit children's engagement with nature. Chief among these barriers are time and scheduling constraints; for instance, a Norwegian study of more than 3,000 parents of children aged 6‐ to 12‐years old indicated that time pressure was the biggest barrier to children spending more time in nature (Skar et al., 2016); similar findings have also been reported in American children (Hofferth, 2009). Physical difficulty accessing natural spaces can also be important (Kellert et al., 2017; Moss, 2012). The first UK lockdown removed time constraints but limited travel out of the immediate area. This unique context provides an opportunity to better understand how some children's connection to nature may respond in the absence of certain limitations (e.g. extracurricular activities, social activities and school) or the addition of others (e.g. travel restrictions and virus fears). The absence of barriers related to time and scheduling should enable some children to spend more time in nature, increasing nature connection and reaping the associated benefits. Consistent with this view, Soga et al. (2021) proposed that changes in human–nature connection during the pandemic follow three pathways: opportunity, capability and motivation. The opportunity pathway considers both positive and negative changes to the opportunities available to access and engage with nature, such as increased time availability and changes to natural space access. The capability pathway is dependent on an individual's capacity, both psychologically and physically, to engage with nature during the pandemic. This could be influenced by changes to mental or physical health. The motivation pathway includes an individual's willingness, drive and desire to engage with nature; the authors speculate that some people might be more motivated to spend time in nature to compensate for increased time indoors while others may view natural elements as potential risks. An individual's experience of each of these pathways during the pandemic will be contingent on personal circumstances; for instance, financial or time constraints were especially likely for low‐income families and families with parents who were key workers.

This novel context also allows investigation into associations between pandemic‐related changes in connection to nature and problems of child well‐being, including emotional and behavioural problems. Emotional problems refer to an individual's emotional and psychological state (e.g. anxiety and depression), while behavioural problems encompass conduct issues such as aggression and hostility (Zilanawala et al., 2019). Measuring emotional and behavioural problems during a time of social and personal upheaval, like during the COVID‐19 pandemic, is important as it allows for better understanding of potential factors that might mitigate negative impacts to well‐being.

As noted above, prior to the pandemic, numerous theorists and practitioners voiced concerns about children's growing disconnection from nature (e.g. Bragg et al., 2013; Imai et al., 2018). Understanding whether the pandemic has affected children's connection to nature is therefore an urgent challenge. In particular, examining whether children's connection to nature changed during this period and identifying the drivers of these changes can serve to guide post‐lockdown initiatives to promote children's connection to nature when ‘normal’ life resumes and to address common limitations to nature engagement. Understanding how connection to nature contributed to child well‐being during this time may also influence decisions concerning future lockdowns or pandemic‐related restrictions. The current work advances knowledge through its focus on young children—an understudied group in this research field. Specifically, the current online survey of British parents of children between 3 and 7 years of age sought to answer the following questions:

1. Did the early stages of the pandemic change children's connection to nature? And, if so, what reasons were given for the change?
2. Did a change in connection to nature differ as a function of child sex, family socio‐economic status and family experience of COVID disruption?
3. What are the implications of a change in connection to nature for children's well‐being?

## 2 METHODS

### 2.1 Sample

Data were collected within a larger online survey‐based study, the i‐FAMS‐Covid study, that included participants from six countries (Australia, China, Italy, Sweden, UK and USA). To maximise comparability across families, only UK responses were included in the current analysis. The study was advertised via social media, and the research team also sent emails to parents of young children who had participated in previous studies. To mitigate low initial participation levels from families with low SES, Cambridgeshire schools agreed to send a special invite to the families of children eligible for pupil premium (additional funds paid to the child's school for children experiencing economic disadvantage) that included a £10 online voucher, activated on completion of the survey. Despite the inclusion of low SES families in Cambridgeshire, this study did not reach a wide enough group of families to be considered representative of the experiences of all children in the United Kingdom, particularly those living in very urban areas or in low socio‐economic status situations.

Parents with a child between the ages of 3‐ and 7‐years old responded to the survey with reference to one target child. In this subsample (*n* = 376) of the main UK survey sample (*n_UK_* = 706), limited to parents who responded to at least one of the current study's key questions, parent respondents had a mean age of 37.93 years (*n =* 367, range = 21–55, *SD* = 5.74), 90% of respondents were female (*n* = 338) and 93.3% reported their ethnicity as White (*n* = 280). In total, 52.3% of target children were male (*n* = 195) and the mean age of the children was 6.06 years (*n =* 376, range = 3.86–7.97 years, *SD* = 1.07). As the nature‐related questions were located near the end of a 30‐min survey, the difference between the number of responses from the main survey sample and the current study's subsample of participants is likely due to dropout that is common in longer online surveys (Hoerger, 2010).

### 2.2 Procedure

The study protocol was reviewed by the University of Cambridge Psychology Research Ethics Committee, reference number PRE.2020.050. A Qualtrics survey (see Appendix S1) was distributed through social media channels in the United Kingdom from 29 April 2020 to 6 July 2020. Parents completed a consent form at the start of the survey and were given several opportunities to opt out of the remainder of the survey as they completed it.

### 2.3 Measures and analytic approach

#### 2.3.1 Connection to nature

Our analyses focussed on parental responses to two survey questions: a forced ‘Yes/No’ response to the question ‘Overall, do you think your child's connection to nature has changed?’ and a free‐text justification question ‘If yes, how do you think your child's connection to nature has changed and why?’. In total, 376 parents responded, of whom 372 answered the forced response question and 307 included a text‐based response. We used qualitative content analysis to examine parents’ text‐based answers. This approach seeks to classify and examine patterns in text to determine the frequency that certain classifications, or codes, appear (Krippendorff, 2018; Miles et al., 2019). Two researchers reviewed the first 40 responses and identified 14 common data‐driven categories, which were used to develop a codebook. To capture the richness of the data, some responses were coded in multiple categories. As a result, the numbers do not always add up to 100%. The two researchers independently coded 30% of the responses and applied Cohen's kappa as an index of inter‐rater reliability (cf. O’Connor & Joffe, 2020). Across all codes, average reliability between coders was 0.87, which falls into Landis and Koch's classification of nearly perfect agreement. However, as O’Connor and Joffe note, presenting an average value for reliability can mask lower values. In this case, all kappa values for individual codes were at least above 0.72, which is categorised as substantial agreement. Having established inter‐rater reliability, the remaining responses were coded by individual researchers.

#### 2.3.2 Socio‐economic status

A composite measure of family socio‐economic status was created as the mean Z‐score of parent/caregiver reported highest level of parental education, parent occupation category, spaciousness and number of bedrooms in the family home. A higher score on this composite variable indicated higher socio‐economic status. Parent occupation and the occupation of the other primary parent/caregiver was coded based on categorisations from the United Kingdom's Office for National Statistics Standard Occupational Classification (ONS, 2020a). Spaciousness of the family's residence was reported by parents/caregivers as either ‘small and cramped,’ ‘small but adequate,’ ‘quite spacious’ or ‘very spacious’.

Additionally, parents were also asked if their child was eligible for pupil premium. Sixty parents indicated that their child was eligible for pupil premium, and as expected, these families had a significantly lower score on the composite SES variable (*M* = −0.74, *SD* = 0.56), compared to children ineligible for pupil premium (*M* = 0.11, *SD* = 0.58), *t*(369) = 10.50, *p* < 0.001.

#### 2.3.3 COVID disruption

COVID disruption was determined based on survey responses to the extent to which a family experienced impacts from COVID‐19 in the form of financial strain, impacts to work situation, family conflict and worry; this was based on work by Prime et al. (2020).

#### 2.3.4 Child well‐being

Parents/caregivers completed the Strengths and Difficulties Questionnaire (SDQ; Goodman, 1997) for the target child during the pandemic. The SDQ is a widely used, validated (Stone et al., 2010) and easy to administer measure of children's behaviour. Subscales were combined to create total emotional and behavioural problems scores (Goodman et al., 2010). The emotional problems subscale comprised the emotional symptoms (e.g. worries, often unhappy, many fears) and peer symptoms (e.g. tends to play alone, bullied) subscales. The behavioural problems score comprised the conduct problems (e.g. has temper tantrums, generally obedient) and hyperactivity (e.g. restless, overactive, cannot stay still for long) subscales. Responses are scored based on a 3‐point Likert scale (1 = not true, 2 = somewhat true and 3 = certainly true). In this sample, internal consistencies were good (Cronbach ɑ for behavioural problems = 0.81; emotional problems = 0.76).

## 3 RESULTS

### 3.1 Did the early stages of the pandemic lead to a change in children's connection to nature? What reasons were given for the change?

In total, 236 parents (63.6%) reported a change in their child's connection to nature. Of these, 206 (54.8% of all parents) reported that their child showed increased connection to nature during lockdown, while just 27 (7.2% of all parents) indicated that their child showed a decreased connection to nature (and six parents did not provide a text explanation to clarify the direction of change—these ‘change with no direction given’ responses were excluded from coding). Additionally, three parents answered the forced‐choice question ‘yes,’ they felt a change did occur, but provided text explanations indicating both a decrease and increase in connection to nature and so their responses were coded as both. Free‐text responses by parents who reported no change often indicated that this lack of change reflected their child's high pre‐pandemic connection to nature, confirming that lack of change in connection to nature does not imply a lack of connection to nature.

The most commonly referenced reasons given by parents for elevated or increased connection to nature were as follows: the child's increased enjoyment of nature, which included references to positive affect as a result of time in or near nature (*n* = 78, 25.4%); the child's increased awareness or interest in nature, which included references to increased observation/noticing of nature as well as references to importance of/interest in nature (*n* = 86, 28%); and having more time to spend outdoors (*n* = 83, 27%). One parent, as part of a response that encapsulated all three of these themes, shared that ‘Our lives have slowed down and so we notice the tiny things, the growth in plants from one day to the next. It has been a very weird headspace, viral armageddon (sic) on our doorsteps but simple quiet beauty of the natural world in our back garden. The former very scary, the latter very comforting’.

These reasons were often supported by supplemental explanations for higher (or steady) connection to nature such as the time of year or weather (*n* = 21, 6.8%), spending time planting and gardening (*n* = 48, 15.6%), engaging in physical activity as a family (*n* = 38, 12.4%), spending time in the garden at the family's home (*n* = 47, 15.3%), changes to routine with a positive impact (*n =* 13, 4.2%) and increases in connection to animals (*n* = 8, 2.6%).

Three reasons were given to explain a decrease in connection to nature or no change without reference to consistently high connection to nature. These were (a) a lack of access to the typical natural spaces the family would utilise to spend time outdoors (e.g. being unable to drive to further away woodlands; *n* = 16, 5.2%); (b) changes in routine that meant the family was less able to spend time outside (*n =* 8, 2.5 = 6%); and (c) the child or parent preferring to stay indoors (*n* = 17, 5.5%). One parent said that their child's connection to nature decreased because of ‘less opportunity to visit places like farms, wildlife centres etc and even places which involve being outdoors and enjoying nature like national trust sights (sic). This has affected her mental health I feel’. Of the 17 references to preferring to stay indoors, just three cited child fears of the virus being outside the home and/or feeling unsafe away from home. For instance, one parent shared that, ‘[Nature connection] has decreased, she only feels safe at home and needed bringing home early on the one time we managed to get her to go for a proper walk’. Table 1 gives illustrative quotations for each category.

**TABLE 1:** Frequencies

	Code	*n* (%)	Illustrative quotation
*Did the early stages of the pandemic lead to a change in children's connection to nature?* *n* = 372	Yes ‐ change	236 (63.6%)	‘More connected due to more time to spend with it’
	No ‐ change	135 (36.4%)	‘She has always been very connected to nature and continues to be so. No change’
*If so, did connection to nature increase or decrease?* *n* = 307	Increased	206 (67.1%)	‘Her connection to nature has changed dramatically. She has become really interested in nature...’
	Decreased	27 (8.8%)	‘He is less inclined to choose to venture outside, preferring to stay indoors’
*What does the change look like?* *Categories explaining increase*	Awareness and interest in nature	86 (28%)	‘She has become really interested in nature, animals and birds. She loves looking for nature on her walks and documents what she sees’
	More time	83 (27%)	‘They have had more time to explore things they already enjoyed’
	Enjoyment of nature and positive affect	78 (25.4%)	‘She's always calmer outside’
	Time spent in garden	47 (15.3%)	‘A little through lots of extra time in the garden’
	Planting and gardening	48 (15.6%)	‘[S]he has taken more interest in growing plants for food this year’
	Physical activity	38 (12.4%)	‘She appreciated our runs and walks because she is at home most of the time so when we go out to exercise she loves it’
	Time of year and weather	21 (6.8%)	‘Due to the good weather he has enjoyed being outside. He's always enjoyed being outdoors, but happened more during lockdown due to good weather’
	Changes to routine with positive impact	13 (4.2%)	‘We have always enjoyed walks around our home and trips to National Trust gardens etc., but have been surprised how readily our children have taken to going on almost daily nature walks…’
	Connection to animals/pets	8 (2.6%)	‘We have lots of rescue animals at home, which he loves taking care of, and has a natural affinity for nature (and they in return, do him)’
*Categories explaining decrease*	Preferring to stay indoors	17 (5.5%)	‘He is less inclined to choose to venture outside, preferring to stay indoors. We don't go on as many regular walks in our local park’
	Lack of access to nature	16 (5.2%)	‘Less opportunity to visit places like farms, wildlife centres etc and even places which involve being outdoors and enjoying nature like national trust sights. This has affected her mental health I feel…’
	Changes to routine with negative or no impact	8 (2.6%)	‘She is a real outdoors child and misses the freedom of life pre quarantine’

### 3.2 Did a change in connection to nature differ as a function of child sex, family socio‐economic status and family experience of COVID disruption?

To examine the association between a change in connection to nature and child demographic variables, parents’ responses to the change in connection to nature questions were categorised into a single variable: no change (36.6%), positive change (53.8%) and negative change (6.6%). Parents who indicated a change but did not indicate a direction (*n* = 7) and those who indicated both a positive and negative change (*n* = 3) were excluded.

**FIGURE 1:**
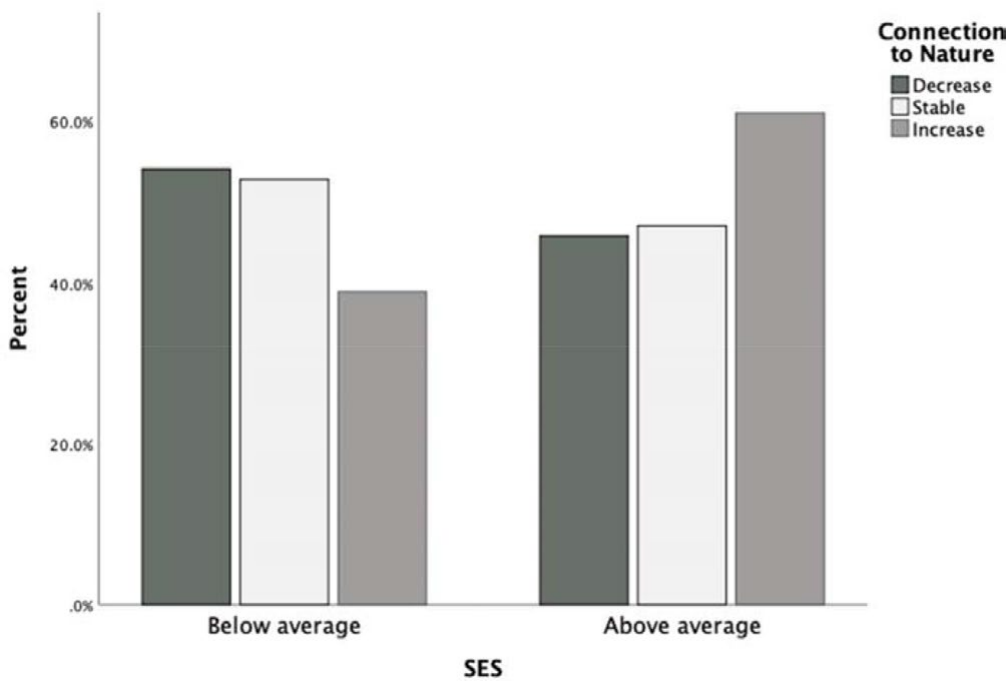
Proportion of children in each connection to nature group that were above or below mean SES (above average *n* = 204, below average *n* = 167)

Neither sex, $\chi_{1}^{2}$ = 3.55, *p* = 0.170, nor extent of COVID disruption, $\chi_{2}^{2}$ = 3.95, *p* = 0.138 were predictive of children's connection to nature. However, both SES, $\chi_{2}^{2}$ = 6.17, *p* = 0.046, and eligibility for pupil premium, $\chi_{2}^{2}$ = 14.96, *p* = 0.001, were predictive, with children from wealthier families being more likely than their less affluent peers to experience increased connection to nature during the pandemic (see Figure 1).

### 3.3 What are the implications of a change in connection to nature for children's well‐being?

**FIGURE 2:**
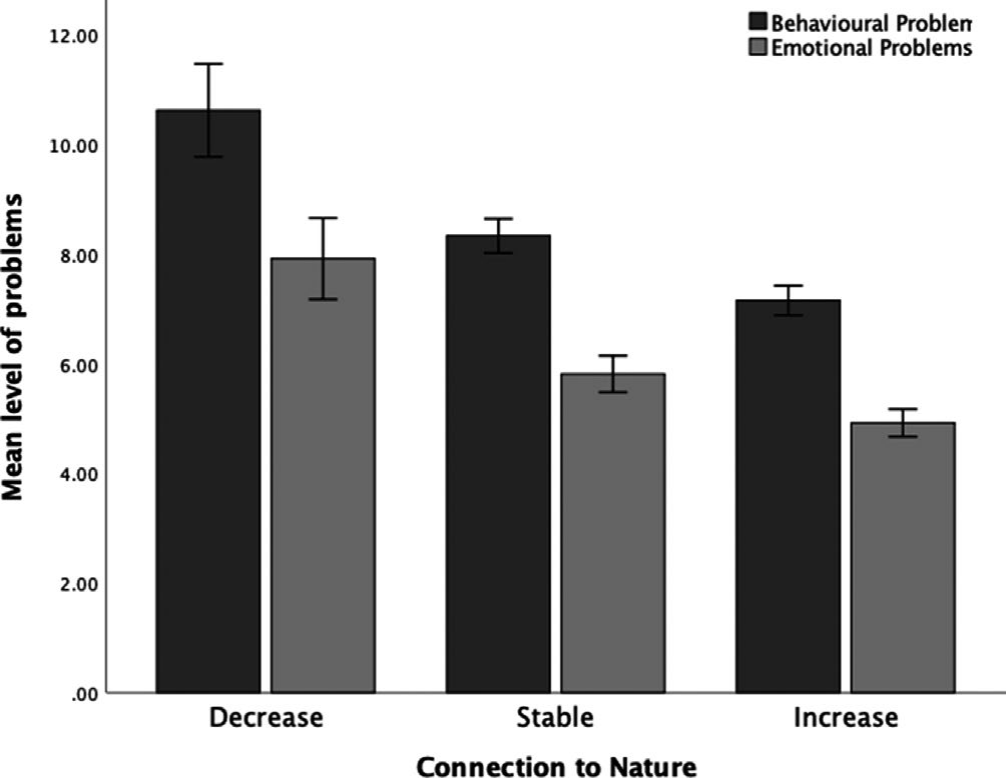
Behavioural and emotional problems as a function of children's changes in connection to nature (error bars = 1 *SE*)

To examine differences in children's behavioural and emotional problems as a function of changes in connection to nature, we conducted an ANCOVA, with SES included as a covariate (see Figure 2). For children's behavioural problems, this analysis showed a significant effect of changes in connection to nature, *F*(2, 357) = 8.82, *p* < 0.001, partial *η* ^2^ = 0.047, even when accounting for SES. Follow‐up pairwise contrasts showed a significantly higher level of behavioural problems in children experiencing either a decrease (*M* difference = 3.14, *p* < 0.01) or stable connection to nature (*M* difference = 0.97, *p* = 0.019) compared with children whose connection to nature increased during the first wave of the pandemic.

A similar pattern was observed for children's emotional problems, such that in addition to a significant effect of SES, there was a significant difference in children's emotional problems as a function of changes in connection to nature, *F*(2, 356) = 6.17, *p* = 0.002, partial *η* ^2^ = 0.033. Follow‐up pairwise contrasts showed a significantly higher level of emotional problems in children experiencing a decrease (*M* difference = 2.65, *p* = 0.001) in connection to nature compared with children who experienced an increase in their connection to nature. However, mean levels of emotional problems did not differ between children experiencing stable compared to increased connection to nature (*M* difference = 0.67, *p* = 0.098). That is, a child who experienced a decrease in their connection to nature was more likely to show emotional problems compared to a child who increased in connection to nature. There was no difference in emotional problems for children who experienced stable connection to nature and those who experienced increased connection to nature.

## 4 DISCUSSION

The COVID‐19 pandemic has provided a novel context through which to study how children's connection to nature has been impacted as well as how these changes may be associated with children's well‐being. Most parents in this UK‐based study reported an increase in their child's connection to nature, and as expected, change in connection to nature was significantly associated with SES. Furthermore, children experiencing an increase in connection to nature were likely to have lower levels of behavioural and emotional problems compared with children whose connection to nature remained the same or decreased during lockdown, even when accounting for SES. We discuss these findings in more detail and their implications for understanding the role of nature for child well‐being below.

Previous studies have identified several barriers to accessing and engaging with nature, many of which are likely to have been altered by the pandemic, including pressures on time (Hofferth, 2009; Skar et al., 2016). We expected more time at home during the pandemic to support increased connection to nature given that this would allow families to spend more time in nature. Consistent with this view, just over a quarter of parents attributed the increased connection to nature to an increased availability of time, supporting existing work on the barriers to accessing and engaging with nature (e.g. Hofferth, 2009; Skar et al., 2016).

Difficulty in accessing natural spaces, a documented barrier (Kellert et al., 2017; Moss, 2012), was reinforced by lockdown restrictions prohibiting travel except for specified reasons and necessitating the closure of many public spaces. Five parents specifically mentioned that their family would typically visit designated natural spaces to engage with nature and strengthen nature connection, but that this was no longer possible during lockdown. Interestingly, however, the lack of access to some spaces necessitated that families change their typical routines and explore local nature. Thus, limitations on accessing physical spaces served a role in both decreasing and increasing nature connection depending on the family's response to that barrier. A high percentage of respondents in the current sample had access to a private garden and so were less affected by the closure of public play spaces, school playing fields and other green spaces than families in urban spaces with no garden access.

The disruption to typical routines, which had both positive and negative implications, allowed for many families to spend more time in nature engaging in a wide variety of activities. Additionally, parents reported that their children demonstrated more interest and awareness of nature as well as increased enjoyment. Enjoyment of nature is often included as a main domain of connection to nature and an important factor in promoting sustainable behaviours in children (Cheng & Monroe, 2012). While less is known about how nature awareness impacts children, increases in noticing nature for adults has been linked to positive affect, psychological well‐being and connection to nature (Lumber et al., 2017; McMahan & Estes, 2015; Passmore & Holder, 2017). Thus, increased enjoyment and awareness of nature seems to lead naturally to increased connection to nature. In many cases, enjoyment and awareness of nature was noted by parents alongside having more time to spend in nature. Based on the responses from this sample, the lockdown period allowed for some children to reconnect with nature. Given the associations between connection to nature and sustainable behaviours (Ives et al., 2018; Whitburn et al., 2019), similar results on a widespread scale would be positive news for the planet and future generations.

Connection to nature was positively associated with SES and more likely in children that were not eligible for pupil premium. That is, children from families with above average SES were more likely than their less affluent peers to have increased in their connection to nature during lockdown. This finding supports recent work with adults demonstrating that during the pandemic, adults from lower SES households in the United Kingdom spent less time outside compared with adults from higher SES households (Burnett et al., 2021). Importantly, and as noted by Oswald et al. (2020), research into access and contact with nature generally draws data from higher SES respondents, while children from lower SES contexts are more likely to engage in higher amounts of screen time and to spend less time in green spaces. It is important to note that while the current study had a relatively homogenous sample of families with respect to SES, we still found differences in connection to nature as a function of SES, suggesting that in a diverse sample these differences would be even more stark.

Access, engagement and connection to nature have well‐documented benefits to well‐being, which is often indexed by the absence of behavioural and emotional problems (Feng & Astell‐Burt, 2017; Maas et al., 2009; Richardson et al., 2017; Sobko et al., 2018; Wells & Evans, 2003). Our findings support those studies conducted prior to the pandemic (e.g. Sobko et al., 2018); specifically, even when accounting for SES, children who increased in their connection to nature over the lockdown displayed fewer behavioural or emotional problems than children whose connection to nature remained stable or deceased. It is important to note, however, that the association between connection to nature and well‐being may be bidirectional in nature, in that parents whose children display elevated behavioural or emotional problems may be less able or willing to arrange outings into natural spaces, thus reducing opportunities for children to increase connection to nature.

In the event of future lockdowns or the re‐introduction of pandemic‐related restrictions, promoting children's connection to nature should be considered as a means of addressing well‐being‐related concerns. For some children, promoting connection to nature may involve encouraging time in the family's garden; for other children, this will not be possible for a variety of reasons, for example having limited access to green space and parental time constraints. Extending the current findings to children living in urban environments and from lower SES families would provide a better understanding of the impact of connection to nature on well‐being in a more representative sample as well as a better understanding of the barriers to facilitating connection to nature faced by families in more diverse situations during the pandemic. It is likely that for many children, the solution will not be as straightforward as heading into the garden.

Alongside the strengths of this work (e.g. the timeliness, novel context and large sample size), this study, like all, has several limitations. First, despite our best efforts to ensure the questionnaire reached a wide audience, our sample was relatively affluent and ethnically homogeneous, with 16.2% of families having a child eligible for pupil premium, below the UK average of 22.6% (ONS, 2020b). Likewise, 93.3% of parents self‐reported their ethnicity as White, as compared with 86% in the last published UK census data (ONS, 2012). Finally, just 5% of respondents stated that they did not have access to a garden, which can be contrasted with national statistics that indicate that as many as 12% of British households did not have access to a garden during the pandemic (ONS, 2020c).

Due to pandemic‐related constraints, we were limited in the methods we could employ to gather data rapidly and safely. As such, we relied on survey methods, which are exclusionary of families who did not have access to the internet during the pandemic (Edwards et al., 2020). The findings of the current study are, therefore, indicative of the experience of a section of the British population. Further work is needed to capture the perspectives of families who live in urban spaces with limited nature access and those who were unable to access the internet during the early pandemic months.

Second, our reliance on parental report, while necessary for work with young children, means that our measure was indirect. The increase parents reported could be a result of parents having more time to notice changes in their children's connection to nature, rather than an actual change in the child's connection to nature. While gathering child perceptions of their own connection to nature is difficult, particularly for younger children who may not be able to accurately self‐report, further research that seeks to capture the perspectives of children in their own words would be valuable.

Third, the first lockdown of the COVID‐19 pandemic in England coincided with exceptionally beautiful spring weather, which may have affected results. That said, fewer than 7% of parents cited the kind weather as a reason for increased connection to nature.

## 5 CONCLUSION

The responses provided by parents to explain the various ways that their children's connection to nature changed during the pandemic can serve to guide future decisions regarding nature access and engagement in the event of further pandemic‐related restrictions as well as in after‐school and educational settings to support a maintenance of higher connection to nature in children. Alongside recommendations such as reducing the number of extracurricular activities for children to allow for more time outside are other actionable changes such as taking part in gardening projects at home and in school. Given a strong evidence base (e.g. Ohly et al., 2016; Waliczek et al., 2000; Wang & MacMillan, 2013), increasing access to materials and land for families to engage in gardening and planting activities could be one way of sustaining increased connection to nature and accessing benefits to well‐being. Distributing funding to allow more schools, particularly those in disadvantaged areas, to implement school gardens and nature‐based learning programs would also support this goal. As the previously noted limitations to accessing nature, particularly those related to time constraints, begin to reappear, learnings from the lockdown era to maintain the increase in connection to nature and general engagement with nature will be needed to ensure that children continue to enjoy opportunities to spend time outdoors. This is also true in the event of future local lockdowns.

Beyond encouraging the increase in connection to nature and working to extend nature connection to children beyond those represented in this sample, the implications that increased connection to nature had for emotional and behavioural problems during this time merit attention. Our analyses suggest that the benefits to child well‐being offered by connection to nature (e.g. Harvey et al., 2020; Richardson et al., 2017; Sobko et al., 2018) applied during the pandemic despite social and personal disruption within families and in the lives of the children in the study. This is important as it demonstrates that the benefits of connection to nature extend in non‐ideal circumstances; promoting nature connection in young children should be considered as a means of promoting well‐being as the effects of the pandemic continue to be felt as well as in the event of future smaller scale difficulties experienced by families. Additionally, the encouraging findings from this study should prompt future work with a more representative sample to determine if these positive effects are indeed observed for children in more diverse circumstances. If not, resources should be allocated to address this discrepancy through increased nature access at home and in school and the implementation of programs that promote nature connection.

Undoubtedly, the COVID‐19 pandemic has led to widespread devastation and loss and has significantly impacted millions of people's well‐being and livelihood. However, this upheaval has also provided many with an opportunity to reflect and to recognise the importance of nature, and children's connection to nature, as a means of addressing increasing mental health problems in young people (Deighton et al., 2019; Terhaag et al., 2021; Waite et al., 2021). It remains to be seen if the increase in children's connection to nature noted in this study will be sustained as lockdown restrictions are eased. Given the importance of connection to nature for well‐being, efforts should be made to maintain this increased connection even after lockdown becomes a distant memory.

## CONFLICT OF INTEREST

The authors declare that there is no conflict of interest.

## AUTHORS' CONTRIBUTIONS

C.H., S.F., E.F. and the i‐FAMS‐Covid study team conceived of the broader survey study idea, designed the methodology and collected the data; S.F., S.I. and E.F. conceived the ideas and methodology for the analysis presented here; S.F. and M.G. conducted the content analysis while E.F. and S.F. conducted the quantitative analysis; S.F. led the writing of the manuscript; S.I., E.F. and C.H. contributed to the drafts; all authors gave final approval for publication.

## Supporting information

Supplementary Material

Appendix S1

## Data Availability

The data supporting the findings of this study are available on the University of Cambridge repository system https://doi.org/10.17863/CAM.75713 (Friedman et al., 2021).
