# Supplementary material for: Understanding changes to children's connection to nature during the COVID‐19 pandemic and implications for child well‐being
Source: People Nat (Hoboken). 2021 Oct 13;4(1):155–65. doi: 10.1002/pan3.10270 (PMC8661645; doi:10.1002/pan3.10270)
Supplement: Supplementary file 1 — Supplementary Material [file PAN3-4-155-s002.pdf]

PEOPLE AND NATURE

Understanding changes to children's connection to nature during the Covid-19 pandemic and impacts for child well-being

Samantha Friedman, Susan Imrie, Elian Fink, Mina Gedikoglu, and Claire Hughes

In the spring and summer of 2020, the Covid-19 pandemic and subsequent lockdowns led to many shifts in daily life, both for better and worse. Families with young children were particularly impacted by these changes as children were expected to complete schooling virtually from home while many parents juggled job responsibilities and other stressors. In addition to the challenges that this time brought, it also provided a unique context to study how children's connection to nature might have been impacted by the disruption to routine.

Psychological connection to nature is a feeling of relatedness or one-ness with nature. Previous researchers have suggested that people who are more connected to nature may act in a way that seeks to protect or help the environment more often. Additionally, children who are more connected to nature may experience benefits to their well-being. To harness these benefits, we felt it was important to understand how children's connection to nature changed during the pandemic and why, as it could help shape post-pandemic initiatives to increase connection to nature and well-being in children.

In order to study this, we conducted a survey study of parents of children aged 3-to-7-years old. Amongst many questions about parent and child relationships and wellbeing, parents were asked to report if their child's connection to nature changed and in what ways. Parents also answered questions about their child's emotions and behaviours.

In our sample of 372 British families, nearly two thirds of parents reported a change (most typically, an increase) in their child's connection to nature. Explanations for this increase included having more time, increased enjoyment of nature, and increased awareness or interest in nature. For these children that increased their connection to nature, they experienced fewer emotional and behavioural problems.

Given that we know it's important to increase connection to nature to encourage pro-environmental behaviours and ensure that children are experiencing the well-being benefits, we feel that efforts should be made to maintain this increased connection during possible future waves of the pandemic as well as after lockdowns and Covid-19 are a distant memory.

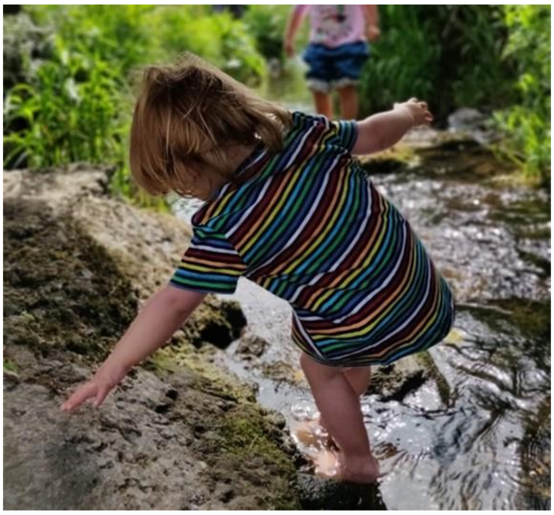

Picture by: Dave Clubb, Unsplash.
