# Supplementary material for: Understanding changes to children's connection to nature during the COVID‐19 pandemic and implications for child well‐being
Source: People Nat (Hoboken). 2021 Oct 13;4(1):155–65. doi: 10.1002/pan3.10270 (PMC8661645; doi:10.1002/pan3.10270)
Supplement: Supplementary file 2 — Appendix S1 [file PAN3-4-155-s001.docx]

Appendix S1 – Copy of survey with relevant questions highlighted on p. 38

Family Impact of COVID-19 - UK

Start of Block: Introduction

Q1   Thank you for taking part in this online survey, which we hope will help policymakers understand the impact of coronavirus quarantine measures on families with young children. The survey is divided into 5 sections.  We estimate that completing the entire survey will take 20-25 minutes on a computer, although completion time is variable. It may take slightly longer to complete on mobile. We’ve included text boxes after each section for you to add comments if you have the time.  Please feel free to take breaks whenever you need! Your progress is automatically saved, so you can click out of the survey and return via the same survey link using the same internet browser whenever you'd like to continue. The survey works best in Firefox, Google Chrome, or Safari.     **Section 1.  COVID, your working life and childcare**   **Section 2.  Health and personal impact of COVID-19**   **Section 3. Your mental health and child adjustment**   **Section 4: Home learning environment**   **Section 5: Your child’s physical activity and contact with nature & screens**   You will find the consent form on the next page.

End of Block: Introduction

Start of Block: Consent form

Q2 Please answer the following questions to consent to participating in the study.

Q3 1. I am an adult with a child aged 3 to 7-years; I have no major psychiatric problems or learning difficulties.

- Yes (1)
- No (2)

Q4 2. I understand that my participation is voluntary and I can opt out of specific questions without consequence.

- Yes (1)
- No (2)

Q156 3. I understand that families with a child who is eligible for pupil premium will be sent a £10 Amazon voucher upon completing the survey.

- Yes (1)
- No (2)

Q5 4. I understand that all data collected will be stored in accordance with General Data Protection Regulation guidelines and the Data Protection act.

- Yes (1)
- No (2)

Q6 5. I understand that because the information about me and my child is anonymous, researchers cannot identify my data and so it is not possible for my responses to be deleted.

- Yes (1)
- No (2)

Q7 6. I understand that anonymous data may be used to support other research and shared with other researchers.

- Yes (1)
- No (2)

Q8 I understand that the research team needs my email address to send me the 6-month follow up survey (which I do not have to complete). I understand that the team will separate my email address from my responses before any analysis, remove it at the end of the study and will not pass it on to any third party. I am happy to be invited to complete the survey again in six months’ time. Please list your preference for contact (email or telephone) and list the relevant email address or phone number.

________________________________________________________________

________________________________________________________________

________________________________________________________________

________________________________________________________________

________________________________________________________________

End of Block: Consent form

Start of Block: Section 1: COVID, Your Working Life, and Childcare

Q103 Please complete the following questions for each adult in the household. We understand that, unfortunately, not all scenarios are represented in the below options. Feel free to explain your specific situation in the text box at the end of this page if you'd like.

Q104 Parent 1 (yourself) - sex

- Male (1)
- Female (2)

Q9 Parent 1 - age

________________________________________________________________

Q105 Parent 1 - educational qualification

- GCSE (1)
- A level/equivalent (2)
- Degree (3)
- Higher degree (4)

Q106 Parent 1 - work situation prior to pandemic

- Full time work (1)
- Part time work (2)
- Not in paid work (3)

Q107 Parent 1 - occupation pre-pandemic

________________________________________________________________

Q108 Parent 1 - income reduction

- None (1)
- 10-20% (2)
- 20-50% (3)
- >50% (4)

Q109 Parent 1 - current work situation

- Working at home - same hours (1)
- Working at home - increased hours (2)
- Working at home - reduced hours (>50%) (3)
- Working at home - very reduced hours ( (4)
- On furlough (5)
- Newly redundant (6)
- Keyworker (7)
- Student (8)
- Unemployed (9)

Q134 If keyworker...role?

________________________________________________________________

Q112 If keyworker...hours?

________________________________________________________________

Q135 If keyworker...contact with COVID patients?

________________________________________________________________

Q114 Please indicate what other adults are in the house (select all that apply).

- Parent 2 (1)
- Other adult 1 (2)
- Other adult 2 (3)
- No other adult (4)

Q115 Parent 2 - sex

- Male (1)
- Female (2)

Q142 Parent 2 - age

________________________________________________________________

Q116 Parent 2 - educational qualification

- GCSE (1)
- A level/equivalent (2)
- Degree (3)
- Higher degree (4)

Q117 Parent 2 - work situation prior to pandemic

- Full time work (1)
- Part time work (2)
- Not in paid work (3)

Q118 Parent 2 - occupation pre-pandemic

________________________________________________________________

Q119 Parent 2 - income reduction

- None (1)
- 10-20% (2)
- 20-50% (3)
- >50% (4)

Q120 Parent 2 - current work situation

- Working at home - same hours (1)
- Working at home - increased hours (2)
- Working at home - reduced hours (>50%) (3)
- Working at home - very reduced hours ( (4)
- On furlough (5)
- Newly redundant (6)
- Keyworker (7)
- Student (8)
- Unemployed (9)

Q139 If Parent 2 is a keyworker...role?

________________________________________________________________

Q140 If Parent 2 is a keyworker...hours?

________________________________________________________________

Q141 If Parent 2 is a keyworker...contact with COVID patients?

________________________________________________________________

Q122 Other adult 1 - sex

- Male (1)
- Female (2)

Q143 Other adult 1 - age

________________________________________________________________

Q123 Other adult 1 - educational qualification

- GCSE (1)
- A level/equivalent (2)
- Degree (3)
- Higher degree (4)

Q124 Other adult 1 - work situation prior to pandemic

- Full time work (1)
- Part time work (2)
- Not in paid work (3)

Q125 Other adult 1 - occupation pre-pandemic

________________________________________________________________

Q126 Other adult 1 - income reduction

- None (1)
- 10-20% (2)
- 20-50% (3)
- >50% (4)

Q127 Other adult 1 - current work situation

- Working at home - same hours (1)
- Working at home - increased hours (2)
- Working at home - reduced hours (>50%) (3)
- Working at home - very reduced hours ( (4)
- On furlough (5)
- Newly redundant (6)
- Keyworker (7)
- Student (8)
- Unemployed (9)

Q136 If other adult 1 is a keyworker...role?

________________________________________________________________

Q137 If other adult 1 is a keyworker...hours?

________________________________________________________________

Q138 If other adult 1 is a keyworker...contact with COVID patients?

________________________________________________________________

Q128 Other adult 2 - sex

- Male (1)
- Female (2)

Q144 Other adult 2 - age

________________________________________________________________

Q129 Other adult 2 - educational qualification

- GCSE (1)
- A level/equivalent (2)
- Degree (3)
- Higher degree (4)

Q130 Other adult 2 - work situation prior to pandemic

- Full time work (1)
- Part time work (2)
- Not in paid work (3)

Q131 Other adult 2 - occupation pre-pandemic

________________________________________________________________

Q132 Other adult 2 - income reduction

- None (1)
- 10-20% (2)
- 20-50% (3)
- >50% (4)

Q133 Other adult 2 - current work situation

- Working at home - same hours (1)
- Working at home - increased hours (2)
- Working at home - reduced hours (>50%) (3)
- Working at home - very reduced hours ( (4)
- On furlough (5)
- Newly redundant (6)
- Keyworker (7)
- Student (8)
- Unemployed (9)

Q110 If other adult 2 is a keyworker...role?

________________________________________________________________

Q121 If other adult 2 is a keyworker...hours?

________________________________________________________________

Q113 If other adult 2 is a keyworker...contact with COVID patients?

________________________________________________________________

Q12 If specified other adults in the home, please indicate here their relationship to the child (adult sibling, aunt, grandparent etc):

________________________________________________________________

Q13 What is your ethnicity?

- White (1)
- Mixed/multiple ethinic groups (2)
- Asian/Asian British (3)
- Black/African/Caribbean/Black British (4)
- Other ethnic group (5)

Q14 If you would like to add any further information, please use this textbox.

________________________________________________________________

| Page Break |  |
| --- | --- |

Q15 Please indicate how many children are in the home:

|  | Please specify number of: | |
| --- | --- | --- |
|  | Boys (1) | Girls (2) |
| 0-3 years (1) |  |  |
| 4-7 years (2) |  |  |
| 8-12 years (3) |  |  |
| 13-17 years (4) |  |  |

Q16 For your child(ren) aged 3-7 years, please give their dates of birth and sex. Please list the target child's information as Child 1. The target child is the child you will mainly report on in the survey. We don't mind which child aged 3-7 you pick as the target child but we'd like this choice to be random, so if you are having trouble deciding which child to focus on, we suggest picking the child whose first name starts with a letter highest in the alphabet:

|  | Sex | Date of birth |
| --- | --- | --- |
|  |  | dd/mm/yyyy (1) |
| Child 1 (target child) (1) | ▼ Male (1 ... Female (2) |  |
| Child 2 (2) | ▼ Male (1 ... Female (2) |  |
| Child 3 (3) | ▼ Male (1 ... Female (2) |  |

Q17 Does anyone in your household have special needs?

- Yes (1)
- No (2)

Q18 If yes, please specify:

________________________________________________________________

Q19 Is anyone in your family eligible for pupil premium?

- Yes (1)
- No (2)

Q102 Do you have any children that are living outside of your household?

- Yes (1)
- No (2)

Q20 How many children are living outside of your household?

|  | Number of children |
| --- | --- |
|  | (1) |
| 0-3 years (1) |  |
| 4-7 years (2) |  |
| 8-12 years (3) |  |
| 13-17 years (4) |  |

Q21 Before the pandemic, how did you divide out-of-school childcare? Use the scale as a percentage 0-100%

|  | Not Applicable |
| --- | --- |

|  | 0 | 10 | 20 | 30 | 40 | 50 | 60 | 70 | 80 | 90 | 100 |
| --- | --- | --- | --- | --- | --- | --- | --- | --- | --- | --- | --- |

| Parent 1 (yourself) () | 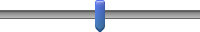 |
| --- | --- |
| Parent 2 () | 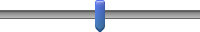 |
| Other adult in the home () | 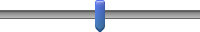 |
| Other adult outside of the home () | 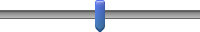 |

Q22 During this quarantine period, how do you divide childcare? Use the scale as a percentage 0-100%

|  | Not Applicable |
| --- | --- |

|  | 0 | 10 | 20 | 30 | 40 | 50 | 60 | 70 | 80 | 90 | 100 |
| --- | --- | --- | --- | --- | --- | --- | --- | --- | --- | --- | --- |

| Parent 1 (yourself) () | 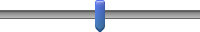 |
| --- | --- |
| Parent 2 () | 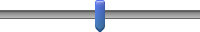 |
| Other adult in the home () | 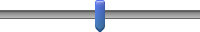 |

| Page Break |  |
| --- | --- |

Q23 How spacious is your home?

- Small and cramped (1)
- Small but adequate (2)
- Quite spacious (3)
- Very spacious (4)

Q24 Please indicate the total number of each type of room in your home.

|  | Total number |
| --- | --- |
|  | (1) |
| Rooms (1) |  |
| Bedrooms (2) |  |
| Bathrooms (3) |  |

Q25 In your home, please estimate the number of:

|  | <5 (1) | 5-10 (2) | 10-20 (3) | >20 (4) |
| --- | --- | --- | --- | --- |
| Toys (1) |  |  |  |  |
| Children's books (2) |  |  |  |  |
| Adult books (3) |  |  |  |  |
| Screens and devices in the home (4) |  |  |  |  |

Q26 Do you have access to an outside space?

- No outside space (1)
- Balcony communal garden (2)
- Small private garden (3)
- Large private garden (4)

Q27 Is your home within walking distance of a green space?

- Yes (1)
- No (2)

Q28 If yes, how often do you make use of this green space?

- At least daily (1)
- Several times a week (2)
- Once a week (3)
- A few times each month (4)

Q29 How well does this green space meet your needs?

- Very well (1)
- Pretty well (2)
- Not very well (3)
- Not at all well (4)

Q30 What is the first part of your Postcode and what country do you live in? (We will use this information to link to other sources of information about the severity and persistence of Covid-19 in your area)

________________________________________________________________

Q31 If you would like to add any further information related to this section, please use the textbox blow

________________________________________________________________

________________________________________________________________

________________________________________________________________

________________________________________________________________

________________________________________________________________

End of Block: Section 1: COVID, Your Working Life, and Childcare

Start of Block: Section 2: Health and Personal Impact of COVID-19

Q32 Has anyone in your household suffered symptoms of COVID-19?

- Yes (1)
- No (2)

Q33 If yes:

|  | Who? | For how long? | How severe? | Are they still affected? |
| --- | --- | --- | --- | --- |
|  |  |  |  |  |
| Please specify: (1) | ▼ Parent 1 (1 ... Child (4) | ▼ Less than 1 week (1 ... More than 2 weeks (3) | ▼ At home (1 ... Intensive care (3) | ▼ Yes (1 ... No (2) |

Q34 Have any of your friends or family needed hospital treatment for Covid-19?

- Yes (1)
- No (2)

Q35 If yes:

|  | Who (relation)? | Did they have intensive care? | Duration of hospital visit: |
| --- | --- | --- | --- |
|  | (1) |  | (1) |
| Please specify: (1) |  | ▼ Yes (1 ... No (2) |  |

Q36 Please indicate the impact this had on you and your family:

|  | Mild (1) | Moderate (2) | Strong (3) | Overwhelming (4) |
| --- | --- | --- | --- | --- |
| Emotional impact (1) |  |  |  |  |
| Practical impact (2) |  |  |  |  |

Q37 Have any of your extended family or close friends passed away as a result of Covid-19?

- Yes (1)
- No (2)

Q38 If yes:

|  | Age | Did they have any underlying health conditions? Please specify: |
| --- | --- | --- |
|  | (1) | (1) |
| Please specify: (1) |  |  |

Q39 Please indicate the impact this had on you and your family

|  | Mild (1) | Moderate (2) | Strong (3) | Overwhelming (4) |
| --- | --- | --- | --- | --- |
| Emotional impact (1) |  |  |  |  |
| Practical impact (2) |  |  |  |  |

Q40 As a result of Covid-19, how much has you or your family experienced:

|  | Not at all (1) | Very little (2) | Moderate (3) | Very much (4) |
| --- | --- | --- | --- | --- |
| Financial strain (6) |  |  |  |  |
| Work/family conflict (7) |  |  |  |  |
| Worry about loved ones (8) |  |  |  |  |

Q41 This question relates to changes in your child's family relationships. Please focus on the target child identified above as aged 3-7 years. Please indicate if there has been a change in closeness between the target child and others in the home:

|  | Marked increase (1) | Small increase (2) | No change (3) | Small decrease (4) | Marked decrease (5) | Not applicable (6) |
| --- | --- | --- | --- | --- | --- | --- |
| With parent 1 (1) |  |  |  |  |  |  |
| With parent 2 (2) |  |  |  |  |  |  |
| With other adults in the home (3) |  |  |  |  |  |  |
| With sibling(s) (4) |  |  |  |  |  |  |
| With adult relatives outside of the home (5) |  |  |  |  |  |  |
| With children outside of the home (cousins/friends/other siblings) (6) |  |  |  |  |  |  |

Q42 This question relates to changes in your child's family relationships. Please focus on the target child identified above as aged 3-7 years. Please indicate if there has been a change in conflict between the target child and others in the home.

|  | Marked increase (1) | Small increase (2) | No change (3) | Small decrease (4) | Marked decrease (5) | Not applicable (6) |
| --- | --- | --- | --- | --- | --- | --- |
| With parent 1 (1) |  |  |  |  |  |  |
| With parent 2 (2) |  |  |  |  |  |  |
| With other adults in the home (3) |  |  |  |  |  |  |
| With sibling(s) (4) |  |  |  |  |  |  |
| With adult relatives outside of the home (5) |  |  |  |  |  |  |
| With children outside of the home (cousins/friends/other siblings) (6) |  |  |  |  |  |  |

| Page Break |  |
| --- | --- |

Q149 Conflict can be healthy, but some behaviours signal relationship problems.
Using the scale below, please indicate how often these occurred **before** Covid-19. 


Me to others

|  | Me to my partner | Me or my partner to other adults in the home |
| --- | --- | --- |
|  |  |  |
| Critcism (1) | ▼ Rarely or never (1 ... Nearly always (5) | ▼ Rarely or never (1 ... Nearly always (5) |
| Controlling (2) | ▼ Rarely or never (1 ... Nearly always (5) | ▼ Rarely or never (1 ... Nearly always (5) |
| Stonewalling (silent treatment) (4) | ▼ Rarely or never (1 ... Nearly always (5) | ▼ Rarely or never (1 ... Nearly always (5) |
| Verbal abuse (5) | ▼ Rarely or never (1 ... Nearly always (5) | ▼ Rarely or never (1 ... Nearly always (5) |
| Pushing/shoving/hitting (6) | ▼ Rarely or never (1 ... Nearly always (5) | ▼ Rarely or never (1 ... Nearly always (5) |

Q43
Using the scale below, please indicate how often these occurred **before** Covid-19.


Others to me or others

|  | My partner to me | Between other adults in the home |
| --- | --- | --- |
|  |  |  |
| Criticism (2) | ▼ Rarely or never (1 ... Nearly always (5) | ▼ Rarely or never (1 ... Nearly always (5) |
| Controlling (3) | ▼ Rarely or never (1 ... Nearly always (5) | ▼ Rarely or never (1 ... Nearly always (5) |
| Stonewalling (silent treatment) (5) | ▼ Rarely or never (1 ... Nearly always (5) | ▼ Rarely or never (1 ... Nearly always (5) |
| Verbal abuse (6) | ▼ Rarely or never (1 ... Nearly always (5) | ▼ Rarely or never (1 ... Nearly always (5) |
| Pushing/shoving/hitting (7) | ▼ Rarely or never (1 ... Nearly always (5) | ▼ Rarely or never (1 ... Nearly always (5) |

Q44 Using the scale below, please indicate how often these occur **since** Covid-19.


Me to others

|  | Me to my partner | Me or my partner to other adults in the home |
| --- | --- | --- |
|  |  |  |
| Criticism (1) | ▼ Rarely or never (1 ... Nearly always (5) | ▼ Rarely or never (1 ... Nearly always (5) |
| Controlling (2) | ▼ Rarely or never (1 ... Nearly always (5) | ▼ Rarely or never (1 ... Nearly always (5) |
| Stonewalling (3) | ▼ Rarely or never (1 ... Nearly always (5) | ▼ Rarely or never (1 ... Nearly always (5) |
| Verbal abuse (4) | ▼ Rarely or never (1 ... Nearly always (5) | ▼ Rarely or never (1 ... Nearly always (5) |
| Pushing/shoving/hitting (5) | ▼ Rarely or never (1 ... Nearly always (5) | ▼ Rarely or never (1 ... Nearly always (5) |

Q152 Using the scale below, please indicate how often these occur **since** Covid-19. 


Others to me or others

|  | My partner to me | Between other adults in the home |
| --- | --- | --- |
|  |  |  |
| Criticism (1) | ▼ Rarely or never (1 ... Nearly always (5) | ▼ Rarely or never (1 ... Nearly always (5) |
| Controlling (2) | ▼ Rarely or never (1 ... Nearly always (5) | ▼ Rarely or never (1 ... Nearly always (5) |
| Stonewalling (4) | ▼ Rarely or never (1 ... Nearly always (5) | ▼ Rarely or never (1 ... Nearly always (5) |
| Verbal abuse (5) | ▼ Rarely or never (1 ... Nearly always (5) | ▼ Rarely or never (1 ... Nearly always (5) |
| Pushing/shoving/hitting (6) | ▼ Rarely or never (1 ... Nearly always (5) | ▼ Rarely or never (1 ... Nearly always (5) |

Q45 The following question relates to changes in relationships with others **outside** the home. Please indicate changes with:

|  | Conflict | Closeness |
| --- | --- | --- |
|  |  |  |
| Extended family (1) | ▼ Much less than before (1 ... Much more (5) | ▼ Much less than before (1 ... Much more (5) |
| Friends (2) | ▼ Much less than before (1 ... Much more (5) | ▼ Much less than before (1 ... Much more (5) |
| Neighbours (3) | ▼ Much less than before (1 ... Much more (5) | ▼ Much less than before (1 ... Much more (5) |
| Child's school (4) | ▼ Much less than before (1 ... Much more (5) | ▼ Much less than before (1 ... Much more (5) |
| Work colleagues (5) | ▼ Much less than before (1 ... Much more (5) | ▼ Much less than before (1 ... Much more (5) |

Q150 The following question relates to changes in relationships with others **outside** the home. Please indicate changes with:

|  | Practical support | Emotional support |
| --- | --- | --- |
|  |  |  |
| Extended family (1) | ▼ Much less than before (1 ... Much more (5) | ▼ Much less than before (1 ... Much more (5) |
| Friends (2) | ▼ Much less than before (1 ... Much more (5) | ▼ Much less than before (1 ... Much more (5) |
| Neighbours (3) | ▼ Much less than before (1 ... Much more (5) | ▼ Much less than before (1 ... Much more (5) |
| Child's school (4) | ▼ Much less than before (1 ... Much more (5) | ▼ Much less than before (1 ... Much more (5) |
| Work colleagues (5) | ▼ Much less than before (1 ... Much more (5) | ▼ Much less than before (1 ... Much more (5) |

| Page Break |  |
| --- | --- |

Q46 How does your 3-7 year old child understand and feel about rules/situation regarding:

|  | Understanding | | | Feelings | | |
| --- | --- | --- | --- | --- | --- | --- |
|  | Often needs reminding (1) | Sometimes needs reminding (2) | Never needs reminding (3) | Often anxious or resistant (1) | Sometimes anxious or resistant (2) | Never anxious or resistant (3) |
| Washing hands often and for 20 seconds (1) |  |  |  |  |  |  |
| Keeping 2 metres apart (2) |  |  |  |  |  |  |
| Staying at home (3) |  |  |  |  |  |  |
| School closure (4) |  |  |  |  |  |  |
| No visits from friends/family (5) |  |  |  |  |  |  |
| Reduced availability of preferred foods (6) |  |  |  |  |  |  |
| Screentime limits (leave blank if n.a.) (7) |  |  |  |  |  |  |

Q47 Is there anything about life under quarantine that your child finds particularly difficult?

- Yes (4)
- No (5)

Q147 If yes, what about life under quarantine has you child found particularly difficult?

________________________________________________________________

________________________________________________________________

________________________________________________________________

________________________________________________________________

________________________________________________________________

Q48 Is there anything about life under quarantine that your child particularly enjoys?

- Yes (4)
- No (5)

Q148 If yes, what about life under quarantine does your child particularly enjoy?

________________________________________________________________

________________________________________________________________

________________________________________________________________

________________________________________________________________

________________________________________________________________

Q49 If you would like to add any further information related to this section, please use the textbox below.

________________________________________________________________

________________________________________________________________

________________________________________________________________

________________________________________________________________

________________________________________________________________

End of Block: Section 2: Health and Personal Impact of COVID-19

Start of Block: Section 3: Your Mental Health and Child Adjustment

Q50 General Health Questionnaire (GHQ) - please complete in reference to your wellbeing. 


In the several weeks **before** the pandemic began, to what extent did the following statements held true for you?

|  |  |  |  |  |
| --- | --- | --- | --- | --- |
| Been able to concentrate on whatever you are doing (Q35_1) | - Better than usual (1) | - Same as usual (2) | - Less than usual (3) | - Much less than usual (4) |
| Lost much sleep over worry (Q35_14) | - Not at all (1) | - No more than usual (2) | - Rather more than usual (3) | - Much more than usual (4) |
| Felt that you are playing a useful part in things (Q35_15) | - More so than usual (1) | - Same as usual (2) | - Less useful than usual (3) | - Much less than usual (4) |
| Felt capable of making decisions about things (Q35_16) | - More so than usual (1) | - Same as usual (2) | - Less so than usual (3) | - Much less capable (4) |
| Felt constantly under strain (Q35_17) | - Not at all (1) | - No more than usual (2) | - Rather more than usual (3) | - Much more than usual (4) |
| Felt you couldn't overcome your difficulties (Q35_18) | - Not at all (1) | - No more than usual (2) | - Rather more than usual (3) | - Much more than usual (4) |
| Been able to enjoy your normal day to day activities (Q35_19) | - More so than usual (1) | - Same as usual (2) | - Less so than usual (3) | - Much less than usual (4) |
| Been able to face up to your problems (Q35_21) | - More so than usual (1) | - Same as usual (2) | - Less so than usual (3) | - Much less than usual (4) |
| Been feeling unhappy and depressed (Q35_22) | - Not at all (1) | - No more than usual (2) | - Rather more than usual (3) | - Much more than usual (4) |
| Been losing confidence in yourself (Q35_23) | - Not at all (1) | - No more than usual (2) | - Rather more than usual (3) | - Much more than usual (4) |
| Been thinking of yourself as a worthless person (Q35_24) | - Not at all (1) | - No more than usual (2) | - Rather more than usual (3) | - Much more than usual (4) |
| Been feeling reasonably happy, all things considered (Q35_25) | - More so than usual (1) | - Same as usual (2) | - Less so than usual (3) | - Much less than usual (4) |

Q51 GHQ continued


Over the last few weeks **during** the quarantine, to what extent have the following statements held true for you?

|  |  |  |  |  |
| --- | --- | --- | --- | --- |
| Been able to concentrate on whatever you are doing (2) | - Better than usual (1) | - Same as usual (2) | - Less than usual (3) | - Much less than usual (4) |
| Lost much sleep over worry (7) | - Not at all (1) | - No more than usual (2) | - Rather more than usual (3) | - Much more than usual (4) |
| Felt that you are playing a useful part in things (8) | - More so than usual (1) | - Same as usual (2) | - Less useful than usual (3) | - Much less than usual (4) |
| Felt capable of making decisions about things (9) | - More so than usual (1) | - Same as usual (2) | - Less so than usual (3) | - Much less capable (4) |
| Felt constantly under strain (10) | - Not at all (1) | - No more than usual (2) | - Rather more than usual (3) | - Much more than usual (4) |
| Felt you couldn't overcome your difficulties (11) | - Not at all (1) | - No more than usual (2) | - Rather more than usual (3) | - Much more than usual (4) |
| Been able to enjoy your normal day to day activities (12) | - More so than usual (1) | - Same as usual (2) | - Less so than usual (3) | - Much less than usual (4) |
| Been able to face up to your problems (13) | - More so than usual (1) | - Same as usual (2) | - Less so than usual (3) | - Much less than usual (4) |
| Been feeling unhappy and depressed (14) | - Not at all (1) | - No more than usual (2) | - Rather more than usual (3) | - Much more than usual (4) |
| Been losing confidence in yourself (15) | - Not at all (1) | - No more than usual (2) | - Rather more than usual (3) | - Much more than usual (4) |
| Been thinking of yourself as a worthless person (16) | - Not at all (1) | - No more than usual (2) | - Rather more than usual (3) | - Much more than usual (4) |
| Been feeing reasonably happy, all things considered (17) | - More so than usual (1) | - Same as usual (2) | - Less so than usual (3) | - Much less than usual (4) |

| Page Break |  |
| --- | --- |

Q52 Your child's adjustment...(please complete for the 3-7 year old target child in your home identified at the start of the survey)

|  | Before quarantine | | | Currently | | |
| --- | --- | --- | --- | --- | --- | --- |
|  | Not true (1) | Somewhat true (2) | Certainly true (3) | Not true (1) | Somewhat true (2) | Certainly true (3) |
| Considerate of other people's feelings (1) |  |  |  |  |  |  |
| Restless, overactive, cannot stay still for long (2) |  |  |  |  |  |  |
| Often complains of headaches, stomach-aches or sickness (3) |  |  |  |  |  |  |
| Shares readily with other children (treats, toys, pencils, etc.) (4) |  |  |  |  |  |  |
| Often has temper tantrums or hot tempers (5) |  |  |  |  |  |  |
| Rather solitary, tends to play alone (6) |  |  |  |  |  |  |
| Generally obedient, usually does what adults request (7) |  |  |  |  |  |  |
| Many worries, often seems worried (8) |  |  |  |  |  |  |
| Helpful if someone is hurt, upset or feeling ill (9) |  |  |  |  |  |  |
| Constantly fidgeting or squirming (10) |  |  |  |  |  |  |
| Has at least one good friend (11) |  |  |  |  |  |  |
| Often fights with other children or bullies them (12) |  |  |  |  |  |  |
| Often unhappy, down-hearted or tearful (13) |  |  |  |  |  |  |
| Generally liked by other children (14) |  |  |  |  |  |  |
| Easily distracted, concentration wanders (15) |  |  |  |  |  |  |
| Nervous or clingy in new situations, easily loses confidence (16) |  |  |  |  |  |  |
| Kind to younger children (17) |  |  |  |  |  |  |
| Often lies or cheats (18) |  |  |  |  |  |  |
| Picked on or bullied by other children (19) |  |  |  |  |  |  |
| Often volunteers to help others (parents, teachers, other children) (20) |  |  |  |  |  |  |
| Thinks things out before acting (21) |  |  |  |  |  |  |
| Steals from home, school, or elsewhere (22) |  |  |  |  |  |  |
| Gets on better with adults than with other children (23) |  |  |  |  |  |  |
| Many fears, easily scared (24) |  |  |  |  |  |  |
| Sees tasks through to the end, good attention span (25) |  |  |  |  |  |  |

Q53 What are your hopes and fears for the next few months regarding your own mental health? Regarding your child's adjustment?

________________________________________________________________

________________________________________________________________

________________________________________________________________

________________________________________________________________

________________________________________________________________

End of Block: Section 3: Your Mental Health and Child Adjustment

Start of Block: Section 4: Home Learning Environment

Q56 **Before** the quarantine, how often did you and your 3 to 7-year old child (identified earlier) engage in the following activities?

|  | Real-life | Screen-based |
| --- | --- | --- |
|  |  |  |
| Picture book/reading (1) | ▼ Did not occur (1 ... N/A (6) | ▼ Did not occur (1 ... N/A (6) |
| Literacy play (e.g. spotting/writing letters of alphabet) (2) | ▼ Did not occur (1 ... N/A (6) | ▼ Did not occur (1 ... N/A (6) |
| Numeracy play (e.g. counting, spotting numbers) (3) | ▼ Did not occur (1 ... N/A (6) | ▼ Did not occur (1 ... N/A (6) |
| Creative play (e.g. arts, crafts, drama, music) (4) | ▼ Did not occur (1 ... N/A (6) | ▼ Did not occur (1 ... N/A (6) |
| Singing/rhymes (e.g. nursery rhymes) (5) | ▼ Did not occur (1 ... N/A (6) | ▼ Did not occur (1 ... N/A (6) |
| Physical activity (e.g. football, dance, Joe Wicks video) (6) | ▼ Did not occur (1 ... N/A (6) | ▼ Did not occur (1 ... N/A (6) |

Q57 **Since** the quarantine started, how often have you and your 3 to 7-year old child engaged in the following activities?

|  | Real life | Screen-based |
| --- | --- | --- |
|  |  |  |
| Picture book/reading (1) | ▼ Did not occur (1 ... N/A (6) | ▼ Did not occur (1 ... N/A (6) |
| Literacy play (e.g. spotting/writing letters of alphabet) (2) | ▼ Did not occur (1 ... N/A (6) | ▼ Did not occur (1 ... N/A (6) |
| Numeracy play (e.g. counting, spotting numbers) (3) | ▼ Did not occur (1 ... N/A (6) | ▼ Did not occur (1 ... N/A (6) |
| Creative play (e.g. arts, crafts, drama, music) (4) | ▼ Did not occur (1 ... N/A (6) | ▼ Did not occur (1 ... N/A (6) |
| Singing/rhymes (e.g. nursery rhymes) (5) | ▼ Did not occur (1 ... N/A (6) | ▼ Did not occur (1 ... N/A (6) |
| Physical activity (e.g. football, dance, Joe Wicks video) (6) | ▼ Did not occur (1 ... N/A (6) | ▼ Did not occur (1 ... N/A (6) |

| Page Break |  |
| --- | --- |

Q58 Thinking back to **before** the COVID-19 pandemic, how often did you do the following activities with your child?

|  | Rarely or never (1) | Monthly (2) | Weekly (3) | Several days per week (7) | Most days per week (4) |
| --- | --- | --- | --- | --- | --- |
| I help my child learn simple sums (e.g. 2+2) (1) |  |  |  |  |  |
| I help my child read words (14) |  |  |  |  |  |
| I encourage my child to do math in his/her head (2) |  |  |  |  |  |
| I ask my child to point to words/letters when we read (3) |  |  |  |  |  |
| We talk about time with clocks and calendars (4) |  |  |  |  |  |
| I introduce new words and their definitions to my child (5) |  |  |  |  |  |
| I help my child weigh, measure, and compare quantities (6) |  |  |  |  |  |
| I help my child to sing/recite the alphabet (7) |  |  |  |  |  |
| We play board games or cards (8) |  |  |  |  |  |
| I encourage collecting (e.g. cards, stamps, rocks) (9) |  |  |  |  |  |
| We make up rhymes in song (e.g. Down by the Bay) (10) |  |  |  |  |  |
| I help my child recite numbers in order (11) |  |  |  |  |  |
| We sing counting songs (e.g. Five Little Monkeys) (12) |  |  |  |  |  |

Q59 How often do you do the following activities with your child **now**?

|  | Rarely or never (1) | Monthly (2) | Weekly (3) | Several days per week (7) | Most days per week (4) |
| --- | --- | --- | --- | --- | --- |
| I help my child learn simple sums (e.g. 2+2) (1) |  |  |  |  |  |
| I help my child read words (14) |  |  |  |  |  |
| I encourage my child to do math in his/her head (2) |  |  |  |  |  |
| I ask my child to point to words/letters when we read (3) |  |  |  |  |  |
| We talk about time with clocks and calendars (4) |  |  |  |  |  |
| I introduce new words and their definitions to my child (5) |  |  |  |  |  |
| I help my child weigh, measure, and compare quantities (6) |  |  |  |  |  |
| I help my child to sing/recite the alphabet (7) |  |  |  |  |  |
| We play board games or cards (8) |  |  |  |  |  |
| I encourage collecting (e.g. cards, stamps, rocks) (9) |  |  |  |  |  |
| We make up rhymes in song (e.g. Down by the Bay) (10) |  |  |  |  |  |
| I help my child recite numbers in order (11) |  |  |  |  |  |
| We sing counting songs (e.g. Five Little Monkeys) (12) |  |  |  |  |  |

| Page Break |  |
| --- | --- |

Q60 Please indicate how well each statement describes your home environment.

|  | Before quarantine | During quarantine |
| --- | --- | --- |
|  |  |  |
| There is very little commotion in our home. (1) | ▼ Very much like our home (1 ... Not at all like our home (4) | ▼ Very much like our home (1 ... Not at all like our home (4) |
| We can usually find things when we need them. (2) | ▼ Very much like our home (1 ... Not at all like our home (4) | ▼ Very much like our home (1 ... Not at all like our home (4) |
| We almost always seem to be rushed. (3) | ▼ Very much like our home (1 ... Not at all like our home (4) | ▼ Very much like our home (1 ... Not at all like our home (4) |
| We are usually able to stay on top of things. (4) | ▼ Very much like our home (1 ... Not at all like our home (4) | ▼ Very much like our home (1 ... Not at all like our home (4) |
| No matter how hard we try, we always seem to be running late. (5) | ▼ Very much like our home (1 ... Not at all like our home (4) | ▼ Very much like our home (1 ... Not at all like our home (4) |
| It's a real zoo in our home. (6) | ▼ Very much like our home (1 ... Not at all like our home (4) | ▼ Very much like our home (1 ... Not at all like our home (4) |
| At home, we can talk to each other without being interuppted. (7) | ▼ Very much like our home (1 ... Not at all like our home (4) | ▼ Very much like our home (1 ... Not at all like our home (4) |
| There is often fuss going at our home. (8) | ▼ Very much like our home (1 ... Not at all like our home (4) | ▼ Very much like our home (1 ... Not at all like our home (4) |
| No matter what our family plans, it usually doesn't seem to work out. (9) | ▼ Very much like our home (1 ... Not at all like our home (4) | ▼ Very much like our home (1 ... Not at all like our home (4) |
| You can't hear yourself think in our home. (10) | ▼ Very much like our home (1 ... Not at all like our home (4) | ▼ Very much like our home (1 ... Not at all like our home (4) |
| I often get drawn into other people's arguments at home. (11) | ▼ Very much like our home (1 ... Not at all like our home (4) | ▼ Very much like our home (1 ... Not at all like our home (4) |
| Our home is a good place to relax. (12) | ▼ Very much like our home (1 ... Not at all like our home (4) | ▼ Very much like our home (1 ... Not at all like our home (4) |
| The telephone takes up a lot of our time at home. (13) | ▼ Very much like our home (1 ... Not at all like our home (4) | ▼ Very much like our home (1 ... Not at all like our home (4) |
| The atmosphere in our home is calm. (14) | ▼ Very much like our home (1 ... Not at all like our home (4) | ▼ Very much like our home (1 ... Not at all like our home (4) |
| First thing in the day, we have a regular routine. (15) | ▼ Very much like our home (1 ... Not at all like our home (4) | ▼ Very much like our home (1 ... Not at all like our home (4) |

Q61 Please use this textbox if you would like to add any further information on this section.

________________________________________________________________

________________________________________________________________

________________________________________________________________

________________________________________________________________

________________________________________________________________

End of Block: Section 4: Home Learning Environment

Start of Block: Section 5: Your Child's Physical Activity and Contact with Nature and Screens

Q63 Please answer for the 3-7 year old child identified at the start of the survey.

Q64 Connectedness to Nature Index

|  |  | | | | |
| --- | --- | --- | --- | --- | --- |
|  | Disagree (1) | Slightly disagree (2) | Neutral (3) | Slightly agree (4) | Agree (5) |
| My child likes to see wild flowers in nature. (1) |  |  |  |  |  |
| Being in nature makes my child feel peaceful. (2) |  |  |  |  |  |
| My child likes to garden and plant. (3) |  |  |  |  |  |
| My child enjoys collecting rocks and shells. (4) |  |  |  |  |  |
| My child feels sad when wild animals are hurt. (5) |  |  |  |  |  |
| My child enjoys touching animals and plants. (6) |  |  |  |  |  |
| My child believes that picking up trash on the ground can help nature. (7) |  |  |  |  |  |
| My child notices wildlife wherever he/she is. (8) |  |  |  |  |  |
| My child chooses to read about plants and animals. (9) |  |  |  |  |  |
| My child is distressed when he/she sees/hears animals being hurt. (10) |  |  |  |  |  |
| My child is heartbroken when animals pass away. (11) |  |  |  |  |  |
| My child is unhappy when the plants are dying. (12) |  |  |  |  |  |
| My child enjoys recycling paper and bottles. (13) |  |  |  |  |  |
| My child feels the difference between outdoors and indoors. (14) |  |  |  |  |  |
| My child reflects/knows that food comes from nature. (15) |  |  |  |  |  |
| My child hears birds and other sounds in nature. (16) |  |  |  |  |  |

Q65 Compared with life before the quarantine, my child...

|  | Outdoors | | | | Indoors | | | |
| --- | --- | --- | --- | --- | --- | --- | --- | --- |
|  | Disagree strongly (1) | Disagree somewhat (2) | Agree somewhat (3) | Agree strongly (4) | Disagree strongly (1) | Disagree somewhat (2) | Agree somewhat (3) | Agree strongly (4) |
| ...has spent more time (1) |  |  |  |  |  |  |  |  |
| ...has been more physically active (2) |  |  |  |  |  |  |  |  |
| ...has shown more enjoyment (3) |  |  |  |  |  |  |  |  |
| ...has appeared more relaxed (4) |  |  |  |  |  |  |  |  |

Q145 Overall, do you think your child's physical fitness has changed?

- Yes (1)
- No (2)

Q66 If yes, how do you think your child's physical fitness has changed and why?

________________________________________________________________

________________________________________________________________

________________________________________________________________

________________________________________________________________

________________________________________________________________

Q146 Overall, do you think your child's connection to nature has changed?

- Yes (1)
- No (2)

Q67 If yes, how do you think your child's connection to nature has changed and why?

________________________________________________________________

________________________________________________________________

________________________________________________________________

________________________________________________________________

________________________________________________________________

Q68 How important do you think these changes will be in the long term?

________________________________________________________________

________________________________________________________________

________________________________________________________________

________________________________________________________________

________________________________________________________________

| Page Break |  |
| --- | --- |

Q69 Finally, we would like to ask about your child's use of screens, which may have changed drastically since the quarantine. For each question, please answer with regards to both before and since the pandemic.

Q70 On a typical day, how much time does your child spend in front of a screen?

Q71 **Before** the pandemic...

|  | 0 mins / N/A (1) |  | 15-30 mins (3) | 30-60 mins (4) | 1-2 hours (5) | >2 hours (6) |
| --- | --- | --- | --- | --- | --- | --- |
| TV/film (1) |  |  |  |  |  |  |
| Games (2) |  |  |  |  |  |  |
| Learning apps (3) |  |  |  |  |  |  |
| Social contact: family (4) |  |  |  |  |  |  |
| Social contact: friends (5) |  |  |  |  |  |  |

Q72 **Since** the pandemic...

|  | 0 mins / N/A (1) |  | 15-30 mins (3) | 30-60 mins (4) | 1-2 hours (5) | >2 hours (6) |
| --- | --- | --- | --- | --- | --- | --- |
| TV/film (1) |  |  |  |  |  |  |
| Games (2) |  |  |  |  |  |  |
| Learning apps (3) |  |  |  |  |  |  |
| Social contact: Family (4) |  |  |  |  |  |  |
| Social contact: friends (5) |  |  |  |  |  |  |

Q73 For each type of screen use, please indicate the main purpose.

Q74 **Before** the pandemic...

|  | Entertaining (1) | Educational (2) | Calming (3) | Sociable (4) | A break for me or other adults (5) |
| --- | --- | --- | --- | --- | --- |
| TV/film (1) |  |  |  |  |  |
| Games (2) |  |  |  |  |  |
| Learning apps (3) |  |  |  |  |  |
| Social contact: family (4) |  |  |  |  |  |
| Social contact: friends (5) |  |  |  |  |  |

Q75 **Since** the pandemic...

|  | Entertaining (1) | Educational (2) | Calming (3) | Sociable (4) | A break for me or other adults (5) |
| --- | --- | --- | --- | --- | --- |
| TV/film (1) |  |  |  |  |  |
| Games (2) |  |  |  |  |  |
| Learning apps (3) |  |  |  |  |  |
| Social contact: family (4) |  |  |  |  |  |
| Social contact: friends (5) |  |  |  |  |  |

Q76 Most of the time your child is engaging with media, who are they watching with?

Q77 **Before** the pandemic...

|  | Alone (1) | With sibling or friend (2) | With adult (3) | Whole family (4) |
| --- | --- | --- | --- | --- |
| TV/film (1) |  |  |  |  |
| Games (2) |  |  |  |  |
| Learning apps (3) |  |  |  |  |
| Social contact: family (4) |  |  |  |  |
| Social contact: friends (5) |  |  |  |  |

Q78 **Since** the pandemic...

|  | Alone (1) | With sibling or friend (2) | With adult (3) | Whole family (4) |
| --- | --- | --- | --- | --- |
| TV/film (1) |  |  |  |  |
| Games (2) |  |  |  |  |
| Learning apps (3) |  |  |  |  |
| Social contact: family (4) |  |  |  |  |
| Social contact: friends (5) |  |  |  |  |

Q79 Please use this textbook if you could like to add any further information on this section.

________________________________________________________________

________________________________________________________________

________________________________________________________________

________________________________________________________________

________________________________________________________________

Q80 Are you happy to finish the survey and have your data collected?

- Yes (1)
- No (2)

End of Block: Section 5: Your Child's Physical Activity and Contact with Nature and Screens

Start of Block: Opt out end

Q81 Are you sure you would like your data deleted?

- Yes (1)
- No (2)

End of Block: Opt out end

Start of Block: OPT OUT YES

Q82 Thank you for your participation. We appreciate your time. Please email covidimpact@psychol.cam.ac.uk if you have any questions.

End of Block: OPT OUT YES

Start of Block: OPT OUT

Q55 Are you sure?

- Yes (1)
- No (2)

End of Block: OPT OUT
